# Post-treatment of hyaluronan to decrease the apoptotic effects of carprofen in canine articular chondrocyte culture

**DOI:** 10.7717/peerj.8355

**Published:** 2020-01-29

**Authors:** Korakot Nganvongpanit, Thippaporn Euppayo, Puntita Siengdee, Kittisak Buddhachat, Siriwadee Chomdej, Siriwan Ongchai

**Affiliations:** 1 Animal Bone and Joint Research Laboratory, Department of Veterinary Biosciences and Public Health, Faculty of Veterinary Medicine, Chiang Mai University, Chiang Mai, Thailand; 2 Excellence Center in Veterinary Bioscience, Chiang Mai University, Chiang Mai, Thailand; 3 Functional Genome Analysis Research Unit, Leibniz Institute for Farm Animal Biology (FBN), Institute of Genome Biology, Dummerstorf, Germany; 4 Department of Biology, Faculty of Science, Naresuan University, Phitsanulok, Thailand; 5 Department of Biology, Faculty of Science, Chiang Mai University, Chiang Mai, Thailand; 6 Department of Biochemistry, Faculty of Medicine, Chiang Mai University, Chiang Mai, Thailand

**Keywords:** Apoptosis, Carprofen, Hyaluronic acid, Cell, Chondrocyte

## Abstract

A major concern associated with the use of drugs is their adverse side effects. Specific examples of the drugs of concern include antibiotic agents and non-steroidal anti-inflammatory drugs. Despite the presence of a high degree of efficacy for specific conditions, these drugs may deteriorate the surrounding tissues that are exposed to them. Often, carprofen is used for joint inflammation; however, it may stimulate cartilage degradation which can then lead to osteoarthritis progression. In this study, hyaluronan was combined with carprofen treatment in three different applications (pre-treatment, co-treatment and post-treatment) on normal canine chondrocytes to determine whether Hyaluronan (HA) is capable of mitigating the degree of chondrotoxicity of carprofen. Our findings revealed that carprofen at IC_20_ (0.16 mg/mL) decreased viability and increased nitric oxide (NO) production. Importantly, carprofen induced the apoptosis of canine chondrocytes via the up-regulation of *Bax*, *Casp3*, *Casp8*, *Casp9* and *NOS2* as compared to the control group. Although the co-treatment of HA and carprofen appeared not to further alleviate the chondrotoxicity of carprofen due to the presence of a high number of apoptotic chondrocytes, post-treatment with HA (carprofen treatment for 24 h and then changed to HA for 24 h) resulted in a decrease in chondrocyte apoptosis by the down-regulation of *Bax*, *Casp3*, *Casp8*, *Casp9*, *NOS2*, along with NO production when compared with the treatment of carprofen for 48 h (*P* < 0.05). These results suggest that HA can be used as a therapeutic agent to mitigate the degree of chondrotoxicity of carprofen.

## Introduction

Several drugs, such as local anesthetic drugs (10 mg/mL of lidocaine or 7.5 mg/mL of ropivacaine in human chondrocyte culture), corticosteroids (25 μm of dextramethasone, 25 mg/mL of prednisolone or 7 mg/mL of betamethasone in human chondrocyte culture), cefazolin (2.0–3.33 mg/mL in canine chondrocyte culture) and fluoroquinolones (182 μg/mL of enrofloxacin or 295 μg/mL of marbofloxacin), have been shown to produce adverse effects on articular cartilage including apoptosis and the inhibition of growth of chondrocyte cells (Chrysis et al., 2005; Farkas et al., 2010; Nakazawa et al., 2002; Siengdee et al., 2016; Siengdee et al., 2017). This phenomenon can result in a loss of extra-cellular matrix formation and the progressive degradation of articular cartilage (Farkas et al., 2010). For example, antibiotics such as cefazolin (2.0–3.33 mg/mL) may cause apoptosis and necrosis of chondrocytes by inducing the alpha tumor necrosis factor (TNF-α) and matrix metelloproteinase (MMP) production, decreasing the expression of key matrix molecules of the cartilage encoded by aggrecan (ACAN) and type II collagen COL2A1, and by downregulating the tissue inhibitors of the matrix metalloproteinase-1 (TIMP1) (Siengdee et al., 2017). Fluoroquinolone treatment has also been widely associated with certain side effects, including the development of severe musculoskeletal disorders, for example, tendinitis and tendon ruptures in humans after a 1-week course of ciprofloxacin, 250 mg twice daily (Akali & Niranjan, 2008), cartilage deformities after receiving ofloxacin, 20 mg/kg once daily for 7 consecutive days in 3-month-old male beagle dogs (Yabe et al., 1997), along with the altering of inflammatory mediators and extracellular matrix (ECM) components, as well as the MMP encoding genes in chondrocyte cultures in vitro (Siengdee et al., 2016). Non-steroidal anti-inflammatory drugs (NSAIDs), including indomethacin, ketorolac, diclofenac, piroxicam and celecoxib, caused proliferation suppression and the cell death of chondrocyte culture in vitro (Chang et al., 2006).

Carprofen (CAR); C_15_H_12_ClNO_2_, belongs to a class of proprionic acid NSAIDs. It is a selective cyclo-oxygenase-2 (COX-2) inhibitor that has been widely used in osteoarthritis treatments in dogs (Monteiro-Steagall, Steagall & Lascelles, 2013) and other animals, such as horses (Goodrich & Nixon, 2006) and cats (Lascelles et al., 2007). The major therapeutic effects of CAR are to reduce pain, inflammation, lameness and the related pain scores (Curry, Cogar & Cook, 2005; Goodrich & Nixon, 2006; Lascelles et al., 2007; Monteiro-Steagall, Steagall & Lascelles, 2013). However, an adverse effect of CAR on cell survival and proteoglycan synthesis has been identified. Additionally, the induction of hepatic toxicosis in dogs as hepatocellular necrosis, lytic necrosis, and apoptosis can result in a decrease in cell viability in canine chondrocyte cultures (Euppayo et al., 2015). Meanwhile, CAR was found to induce oxidative stress in the mitochondria of the colonic mucosa of dogs (Snow et al., 2014). Moreover, CAR is highly potent and has been proven to be capable of inducing p75^NTR^ dependent apoptosis via the p38 MAPK pathway in prostate cancer cells (Khwaja et al., 2008). A previous study has revealed that CAR could significantly decrease glycosaminoglycan release and elevated proteoglycan synthesis in equine cartilage explants (Armstrong & Lees, 1999). However, the potential side effects of CAR on normal chondrocytes has not been well-established in terms of its toxicity on viability, which has contributed to the current aims of this study.

Hyaluronan (HA), an anionic and non-sulfated glycosaminoglycan, plays an important role in forming and maintaining the structure of the ECM and the synovial fluid in articular cartilage. HA is composed of a repeated disaccharide structure of N-acetylglucosamine and glucuronic acid ((1→3)-β-d-GlcNAc-(1→4)-β-d-GlcA-). Intra-articular HA injection has been recommended by the European Society for Clinical and Economic Aspects of Osteoporosis and Osteoarthritis for patients who do not respond to NSAIDs (Bruyère et al., 2016). HA restores the mechanical visco-supplementation of joints and also supports joint homeostasis by inducing endogenous HA production via synoviocytes (Maheu, Rannou & Reginster, 2016) and by stimulating the proliferation of chondrocytes in vitro (Akmal et al., 2005; Kawasaki et al., 1999). Both in vitro and in vivo studies have shown that HA has the potential to reduce the side effects of some drugs on chondrocytes. Pre-incubation with HA (molecular weight 500–730 kDa) before exposure to interleukin 1 beta (IL-1β) and TNF-α can reduce chondrocyte apoptosis and mitochondrial DNA damage (Grishko et al., 2009; Zhou, Liu & Peng, 2008) and can block nitric oxide (NO)-induced apoptosis (Peng et al., 2010). Moreover, intra-articular injection of HA after trauma could reduce chondrocyte apoptosis in rabbits (Barreto et al., 2015) and dogs (Nganvongpanit et al., 2013). The hypothesis of this study was that HA could reduce the apoptosis effects of drugs on the chondrocyte culture. Our previous studies involving canine chondrocyte cultures in vitro produced a different result when HA was combined with other drugs. However, HA could lessen the side effects of fluoroquinolones when treatment was given in conjunction with those other drugs (Siengdee et al., 2016). On the contrary, the therapeutic effects of HA could not mitigate the side effects of carprofen (Euppayo et al., 2015), corticosteroid (Siengdee et al., 2015) and triamcinolone acetonide (Euppayo et al., 2016) when subjects were treated with HA at the same time.

The purpose of this study was to investigate the potential effects of HA in three different applications; combined, pretreatment and post-treatment with CAR on normal canine chondrocytes by evaluating chondrocyte viability, expression of the apoptosis genes and NO production. In this study, normal chondrocytes were used instead of OA chondrocytes because the normal chondrocyte culture might have mimicked the surrounding tissue and still not be directly affected by the inflammation state or OA pathology, but could be exposed to CAR and possibly be modified.

## Materials and Methods

### Reagents

A commercial low molecular weight hyaluronan (HA) (Maheu, Rannou & Reginster, 2016) 10 mg/mL, 500–750 kDa (TRB Chemedica, Bangkok, Thailand) was used in this study. Additionally, a commercial CAR injection of 50 mg/mL (Zoetis, Bangkok, Thailand) was applied in this study for all experiments.

### Experimental design

Chondrotoxicity of CAR on the chondrocytes was estimated using 3-(4,5-dimethylthiazol-2-yl)-2,5-diphenyltetrazolium bromide (MTT) assay. The experiment firstly used CAR (50% w/v) stock solution that was then diluted in Dulbecco’s modified Eagle’s medium (DMEM; Invitrogen, Waltham, MA, USA) serially for the appropriate drug concentrations (0.05, 0.10, 0.20, 0.39, 0.78, 1.56, 3.13, 6.25, 12.5 and 25 mg/mL). The data was then used to plot graphs of chondrocyte viability and to estimate the concentration of CAR that resulted in 20% cell death after 24 h of treatment. This optimum concentration (IC_20_) of CAR was then selected to be combined with HA at 10 µg/mL. Ten culture conditions were used, and this study was divided into six control groups and four treatment groups that included the following:

Control groups:

1. Culture without treatment for 24 h (Cont24)
2. Culture without treatment for 48 h (Cont48)
3. Chondrocyte treated with CAR for 24 h (CAR24)
4. Chondrocyte treated with CAR for 48 h (CAR48)
5. Chondrocyte treated with HA for 24 h (HA24)
6. Chondrocyte treated with HA for 48 h (HA48)

Treatment groups:

1. Chondrocyte treated with HA and CAR and incubated for 24 h (Comb24)
2. Chondrocyte treated with HA and CAR and incubated for 48 h (Comb48)
3. Chondrocyte treated with HA for 24 h then replaced by CAR for 24 h (Pre-tr)
4. Chondrocyte treated with CAR for 24 h then replaced by HA for 24 h (Post-tr)

At the end of all treatments, the effects of the combinations were assessed for cell viability using MTT assay, while NO production was assessed using Griess reaction. The ensuing expression levels of the genes that were involved in the apoptosis pathway were assessed, and the anabolic and catabolic genes were measured using real-time qPCR.

### Normal canine primary chondrocyte culture

Canine chondrocytes obtained from normal joints (smooth cartilage surface, having no inflammation of the joint capsule with clear and yellowish synovial fluid) were harvested from dog cadavers (*n* = 3) at the Veterinary Cadaveric Unit, Faculty of Veterinary Medicine, Chiang Mai University, Chiang Mai, Thailand. All subjects had previously experienced some type of accident or suffered from some other disease that did not involve the musculoskeletal system. According to the Animals for Scientific Purposes Act, B.E. 2558 (2015), since only a portion of this experiment was performed on animal carcasses, no ethical approval was required for this study. This was confirmed by the Animal Ethics Committee, Faculty of Veterinary Medicine, Chiang Mai University (License Number U1006312558).

The skin and muscle tissues nearest to the joint were removed before opening the joint to collect cartilage using the aseptic technique within 6 h after the animal had died. After pieces of cartilage were collected, an immerged piece of cartilage was washed in PBS. Pieces of cartilage were then chopped into 1–2 mm sections and placed on a petri-dish. The pieces of cartilage were incubated in 10% collagenase type II (Gibco, New York, USA) in DMEM for 21 h at 5% CO_2_, 37 °C and 70% relative humidity. Subsequently, the pieces of cartilage were washed twice with PBS and replaced with growth medium (DMEM supplemented with 10% fetal bovine serum (FBS; Gibco, New York, USA) and 1% antibiotic/antimycotic (Gibco, New York, USA). The explant samples were then cultured in 5% CO_2_ at 37 °C and with 70% relative humidity. The culture medium was changed every 2–3 days. The migration of the primary chondrocyte cells could clearly be seen after 2 weeks. When the primary chondrocyte cells reached ∼80% confluence in the culture flasks, the cells were trypsinized by 0.1% trypsin/EDTA. Consequently, cells in the 3–6 passage were used in this experiment.

### Chondrotoxicity of HA and CAR by MTT assay

In order to find suitable concentrations at IC_20_ of CAR, normal canine articular chondrocytes were trypsinized by 0.1% trypsin/EDTA. Subsequently, 20,000 cells/well were seeded in 100 µL in 96-well-plates and cultured in DMEM that had been supplemented with 5% FBS and incubated at 37 °C under conditions previously described for 24 h before being treated with CAR at 0.05, 0.10, 0.20, 0.39, 0.78, 1.56, 3.13, 6.25, 12.5, 25 mg/mL in triplication. The cells were then further incubated for 24 and 48 h. After the incubation period was over, the media was changed to DMEM containing MTT (0.5 mg/mL) and the cells were further incubated for 4 h. The MTT was then replaced with 100 µL of dimethyl sulfoxide (DMSO) and were mixed well. The absorbance intensity was measured using a microplate reader (Thermo Scientific Multiskan^®^ EX, Vantaa, Finland, Europe) at 490 nm. The percentage of cell viability was determined using the following equation:

$${\rm Percentage\ of\ cell\ viability} = ({\rm Absorbance\ of\ sample/ Absorbance\ of\ control}) \times 100$$

To measure the chondrotoxicity of HA together with CAR (under all treatment conditions; Comb, Pre-tr, and Post-tr), chondrocytes were seeded in 96-well-plates and assessed using MTT assay to determine the percentage of cell viability. This was done after chondrocytes were treated with IC_20_ of CAR in combination with HA at a concentration 10 µg/mL for 24 h. Notably, 10 µg/mL of HA was found to be effective in reducing rat chondrocyte apoptosis that had been induced by IL-1β through a decreased percentage of cells undergoing apoptosis, an increased ATP level of mitochondria, and a reduced level of *NOS2* expression at the prescribed transcription level (Zhou, Liu & Peng, 2008).

### Reverse transcription and real-time qPCR

Chondrocytes were seeded into 6-well-plates at 200,000 cells/well and incubated for 24 h. The cells were then treated with CAR at IC_20_ and 10 µg/mL HA under different treatment conditions. After incubation under each set of conditions, the cells were collected to extract total RNA using innuPREP DNA/RNAMini Kit (Analytik Jena, Jena, Germany) and converted to cDNA by Tetro cDNA Synthesis kit (Bioline, Taunton, MA, USA). Genes as mediators of apoptosis; BCL2 Associated X, Apoptosis Regulator (*BAX*), Apoptosis regulator (*BCL2*), BCL2 Like 1(*BCL2-xL*), Caspase 3 (*CASP3*), Caspase 8 (*CASP8*), Caspase 9 (*CASP9*) and NO synthase 2 (*NOS2*) were assessed in terms of the effect of the drugs on chondrocyte apoptosis (six replicate experiments). Aggrecan (*ACAN)*, collagen type II alpha 1 Chain (*COL2A1*), hyaluronan synthase 1*(HAS1)* and TIMP metallopeptidase inhibitor 1 (*TIMP1*) were used to estimate the effects of the drugs on the chondrocyte anabolism pathway. Genes involved in the catabolism pathway of chondrocytes, including interleukin-1 Beta (*IL-1*β), matrix metalloproteinase**-**2 (*MMP2*) and matrix metalloproteinase**-**9 (*MMP9*), were assessed in terms of their level of expression (four replicate experiments).

The sequence of the primers is listed in Table 1. The qPCR reaction used 2x SensiFAST SYBR^®^ No-ROX Mix (Bioline, England) 5 μl, 10 μm forward and reverse primer 0.2 μl each, and cDNA at 20 ng. The levels of expression of these genes were calculated relative to glyceraldehyde-3-phosphate dehydrogenase (*GAPDH*) using the 2^−ΔΔCt^ method (Heid et al., 1996; Livak & Schmittgen, 2001).

**Table 1:** Primer sequences for gene expression analysis.

Gene symbol	Primer forword (5′→3′)	Primer reverse (5′→3′)	Size (bp)	NCBI Accession Number
*GAPDH*	AGTATGATTCTACCCACGGC	CGAAGTGGTCATGGATGACT	262	NM_001003142.2
Genes related to apoptosis pathway				
*BAX*	GAGAGGTCTTTTTCCGAGTGG	CCTTGAGCACCAGTTTGCTG	105	NM_001003011.1
*BCL2*	TGTGTGTGGAGAGCGTCAA	GACAGCCAGGAGAAATCAAACAG	178	NM_000633.3
*BCL2-xL*	CTGTGCGTGGAAAGCGTAGA	CCAAGGCTCTAGGTGGTCAT	96	NM_001003072.1
*CASP3*	CTCAGGGAAACATTCACAAAC	TAGAAGCACGCAAACAAAAC	138	NM_001003042
*CASP8*	GCTTCAGATACCAGGCAGAG	CAGGCTTTTGTTGATTTGG	247	NM_001048029
*CASP9*	TGGTGGGGAGCAGAAAGA	AGCCTGGGAAGGTGGAATAG	191	NM_001031633
*NOS2*	TCTGTGGGGATGTGCGTATG	CTTGCTCTTCACTCAGGCTCA	87	NM_001313848.1
Genes related to anabolic and catabolic pathway				
*ACAN*	GCCACCATCAGAAACCTAC	AGACACCTCGGAAGCAGA	350	NM_001113455.2
*COL2A1*	CAGCGAGCGTTCCCAAGA	CAGGCGGAGGAAGGTCAT	158	NM_001006951.1
*HAS1*	CAGACACGCTGGTCCAA	GCATAGAAGAGCCGCAACAC	149	XM_022404705.1
*IL-1*β	CACAGGTTCTCTGGTAGATGAGG	TGGCTTATGTCCTGTAACTTGC	264	NM_001037971.1
*MMP2*	CAGGGTCCATAGCTCATCGT	CTGGCTGTGCAATACCTGAA	501	XM_014109407.1
*MMP3*	GGTTGGAGGTGACAGGGAAG	CCAGGGAAGGTGGTGAAGTC	100	NM_001002967.1
*MMP9*	AACGGGCTTCTGGCTCACGC	GCTGCACCAGGGCGTGTCAT	219	NM_001003219.2
*TIMP1*	ATCCTGCTGTTGCTGTGG	GTCGGTCTGGTTGACTTCTGC	138	NM_001003182.1

**Note:**

*ACAN*, aggrecan; *BAX*, BCL2 Associated X; *Bcl-2*, B-cell lymphoma 2; *BCL-xL*, Bcl*-*2*-*Like Protein 1; *CASP3*, Caspase 3; *CASP8*, Caspase 8; *CASP9*, Caspase 9; *COL2A1*, collagen type II alpha 1; *GAPDH*, glyceraldehyde-3-phosphate dehydrogenase; *HAS1*, hyaluronan synthase 1; *IL-1*β, interleukin*-*1β; *MMP2*, matrix metalloproteinase**-**2; *MMP3*, matrix metalloproteinase**-**3; *MMP9*, matrix metalloproteinase**-**9; *NOS2*, nitric oxide synthase; *TIMP1*, TIMP metallopeptidase inhibitor 1.

### Hoechst staining for analysis of chondrocyte apoptosis

Chondrocytes were seeded at 50,000 cells/well on 24-well-plates that were comprised of a round cover slip on the bottom of each well. Chondrocytes were then incubated for 24 h. The drug treatments of the 10 groups were examined as has been described above using nine replicate experiments. The treated chondrocytes in each well were then fixed with 4% paraformaldehyde overnight. The cells were washed with PBS and the cells were then incubated with 40 μm Hoechst dye No. 33342 (Invitrogen, Waltham, MA, USA) in PBS for 15 min in the dark and at room temperature. The stained cells on each cover slip were removed and placed on a slide. Twenty pictures of each experimental group were randomly taken using a fluorescence microscope at 330–385 nm (Olympus, Japan). The pictures of the normal chondrocytes and the apoptotic chondrocytes (nuclear condensation and fragmented nuclei) were manually counted using the cell counter in the ImageJ program. The percentage of chondrocyte apoptosis was estimated using the following equation:

$${\text{Apoptotic cell}}\ (\%) = ({\text{total number of apoptotic cells/total number of cells}})\times 100$$

### NO production measurement for analysis of nitrites

The medium for our culture experiments using different strategies of HA and CAR treatment at 24 and 48 h were collected. Firstly, 150 µL of each sample was pipetted into each well. Standard NaNO_2_ diluted with DMEM (0, 1.56, 3.13, 6.25, 12.5, 25, 50 and 100 µm) was added into each well of the 96-well-plates in duplicate. Then, 150 µL of Griess reagent (modified) (Sigma, St. Louis, MO, USA) was added to each well and the specimens were gently mixed at 450 rpm for 1 min. After that, the plates were immediately measured in terms of optical density (OD) using a microplate reader set at 540 nm. The concentrations of nitrite in the culture medium samples were calculated from the standard graph plot of the NaNO_2_ concentrations and (*X*-axis) and OD at 540 nm (*Y*-axis).

### Statistical analysis

The differences in chondrocyte viability, apoptosis, NO production and the gene expression profile of each treatment were assessed using one-way analysis of variance (ANOVA). Additionally, Tukey’s test was used for multiple comparisons. The data of these tests are presented in mean ± standard deviation in bar graphs that had been generated under R program version 3.6.0.

## Results

### Chondrotoxicity of CAR

**Figure 1:**
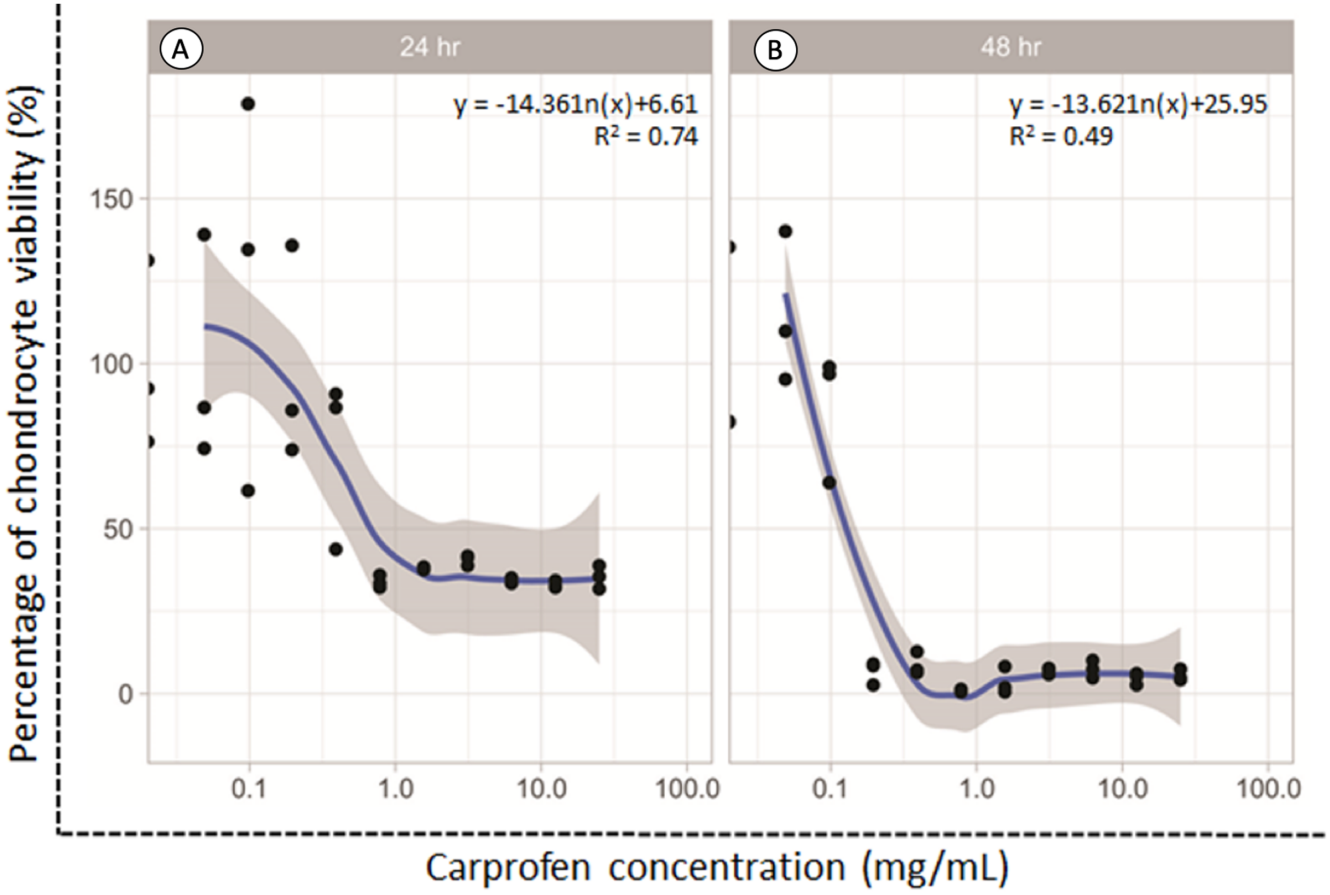
Percent viability of canine derived normal articular cartilage chondrocytes after treatment with two-fold dilutions of carprofen (CAR) at 24 h (A) and 48 h (B) of incubation. This experiment was performed in three replications.

The dose responsive effects of CAR on chondrocyte viability after being treated for 24 and 48 h were determined by MTT assay. The percentage of cell viability was found to be concentration-dependent inversely in relation to the CAR concentrations after both the 24 and 48 h treatments (Fig. 1). The average doses of CAR at IC_20_ at 24 and 48 h were found to be 0.16 mg/mL and 0.02 mg/mL, respectively. These doses were used to induce chondrotoxicity and chondrocyte apoptosis in all experiments throughout this study.

### Effect on chondrocyte viability, chondrocyte apoptosis and NO production

**Figure 2:**
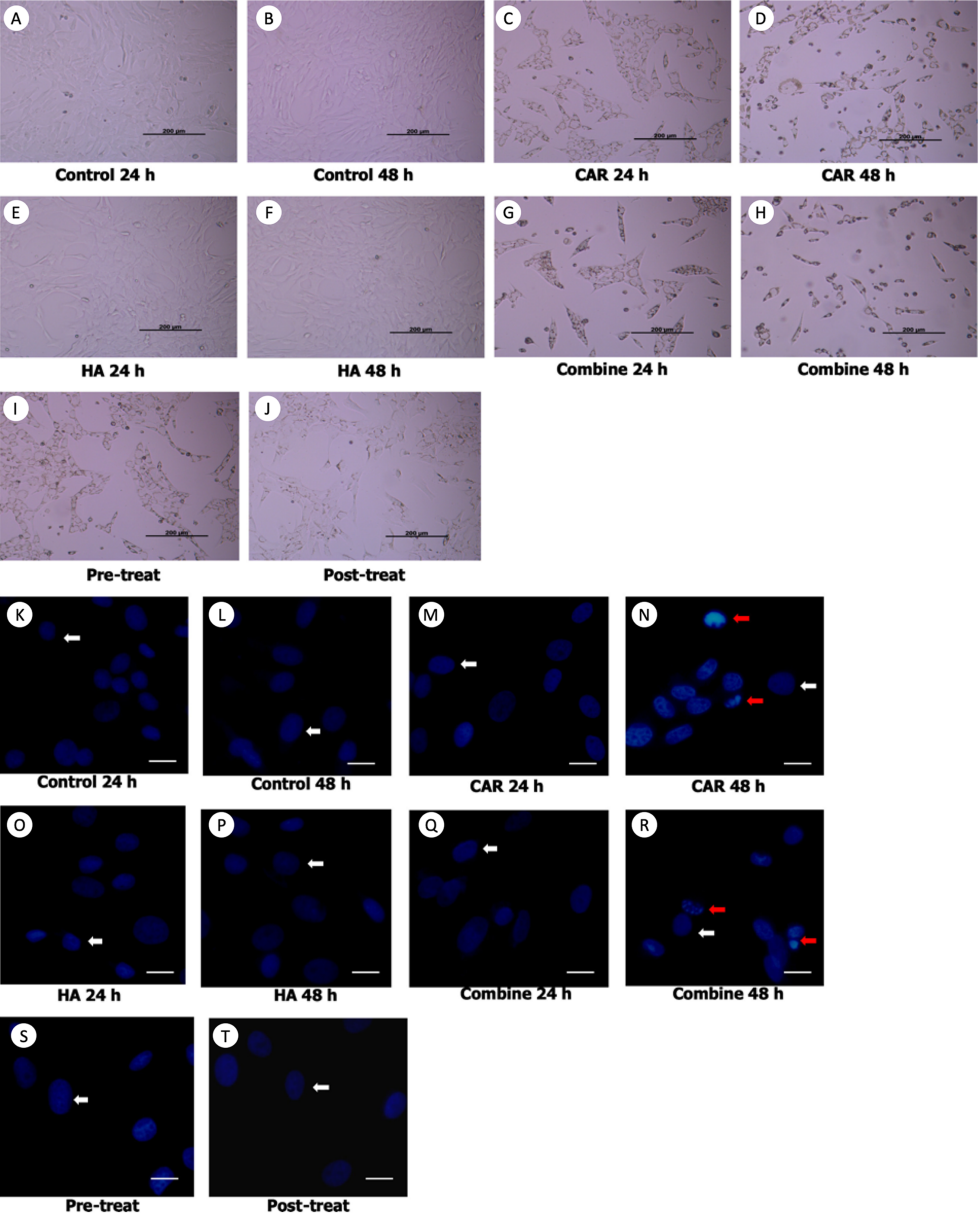
Chondrocyte morphology (A–J) and apoptotic chondrocytes (K–T) after being treated with carprofen (CAR) at IC_20_, hyaluronic acid (HA) 10 µg/ml under different conditions (combine, pre-treat, and post-treat) at 24 h and 48 h of incubation. Chondrocyte morphology (A–J) was observed using a light microscope; the bar indicates 200 µm. Images of normal and apoptotic chondrocytes (K–T) in all experimental groups after being stained by Hoechst dye. As determined by fluorescent microscope, the bar indicates 10 µm (A). Nucleus of normal treated chondrocytes is shown in blue color (white arrows). Apoptotic chondrocytes are shown in light blue color of nuclear condensation, while apoptotic bodies appear as nuclear fragmentation (red arrows).

**Figure 3:**
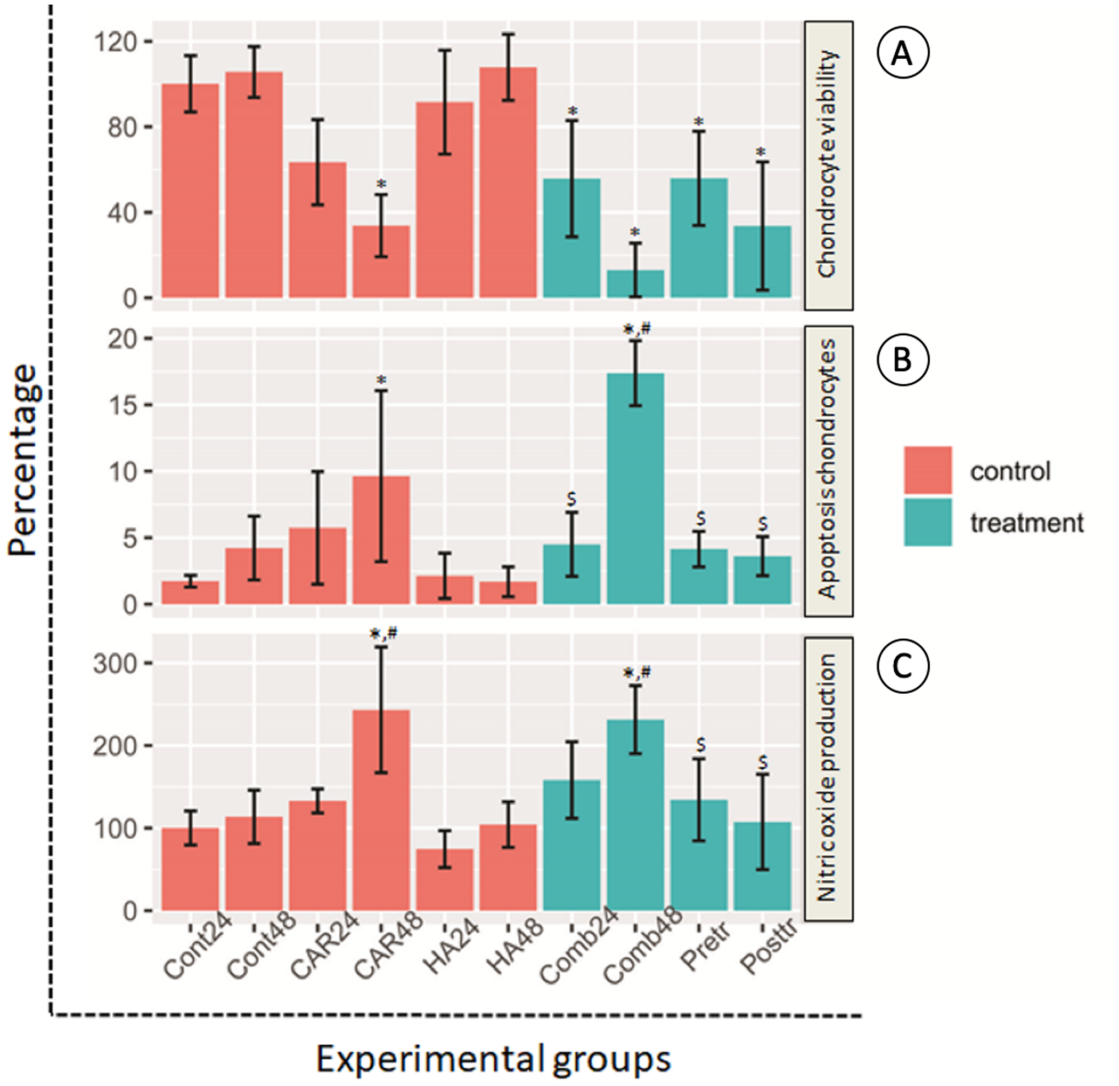
Chondrocyte viability (A), apoptotic chondrocytes (B) and nitric oxide production (C) of normal chondrocytes insix control (orange bars) and four treatment groups (turquoise bars). Ten treatment groups are presented and are comprised of the control (Cont) at 24 h and 48 h of incubation; the group treated with carprofen (CAR) and hyaluronic acid (HA) and the group treated with a combination (Comb) of CAR and HA at 24 h and 48 h of incubation; and the pretreatment (Pre-tr) and post-treatment (Post-tr) groups at 48 h of incubation. This experiment was performed in nine replications and significant differences were identified when *P* < 0.05): “*” represents significant differences when compared with the Cont24 and Cont48 groups, “#” represents significant differences when compared with the CAR24 group and “$” represents significant differences when compared with the CAR48 group.

Morphological changes of the treated cells were observed under a phase contrast inverted microscope. Hoechst staining was done using a fluorescence microscope and results are exhibited in Figs. 2A and 2B. It was observed that after the chondrocytes were treated with CAR at IC_20_ alone or with a co-treatment of HA under every condition (combined, pretreated, and post-treated) at both 24 and 48 h of incubation, the cells changed in terms of cell morphology. Cells were determined to have undergone apoptosis by displaying apoptotic bodies, a decrease in cell numbers, and a loss of cellular adhesion when they eventually detached from the culture surface and appeared to be floating in the culture medium. Chondrocyte viability (Fig. 3A) of the CAR48 group was significantly lower than that of the Control and HA groups (both at 24 and 48 h of culturing). Combinations of HA and CAR under all conditions (Comb 24, Comb 48 h, Pre-tr and Post-tr) were found to significantly reduce the percentage of chondrocyte viability when compared with both the Control and HA groups at 24 and 48 h of incubation. The percentages of apoptotic chondrocytes are shown in Fig. 3B. CAR treatment alone and the Comb group at 48 h displayed a significantly higher average percentage of apoptotic value than the other groups. The percentage of NO production is shown in Fig. 3C. As is shown in Fig. 3C, NO production of both CAR and Comb groups at 48 h were found to be significantly higher than that of the CAR 24, Pre-tr, Cont (24 and 48 h) and HA (24 and 48 h) groups. However, significant differences were not observed in the other groups.

### Effect on mRNA expression

#### Genes related to apoptosis pathway

**Figure 4:**
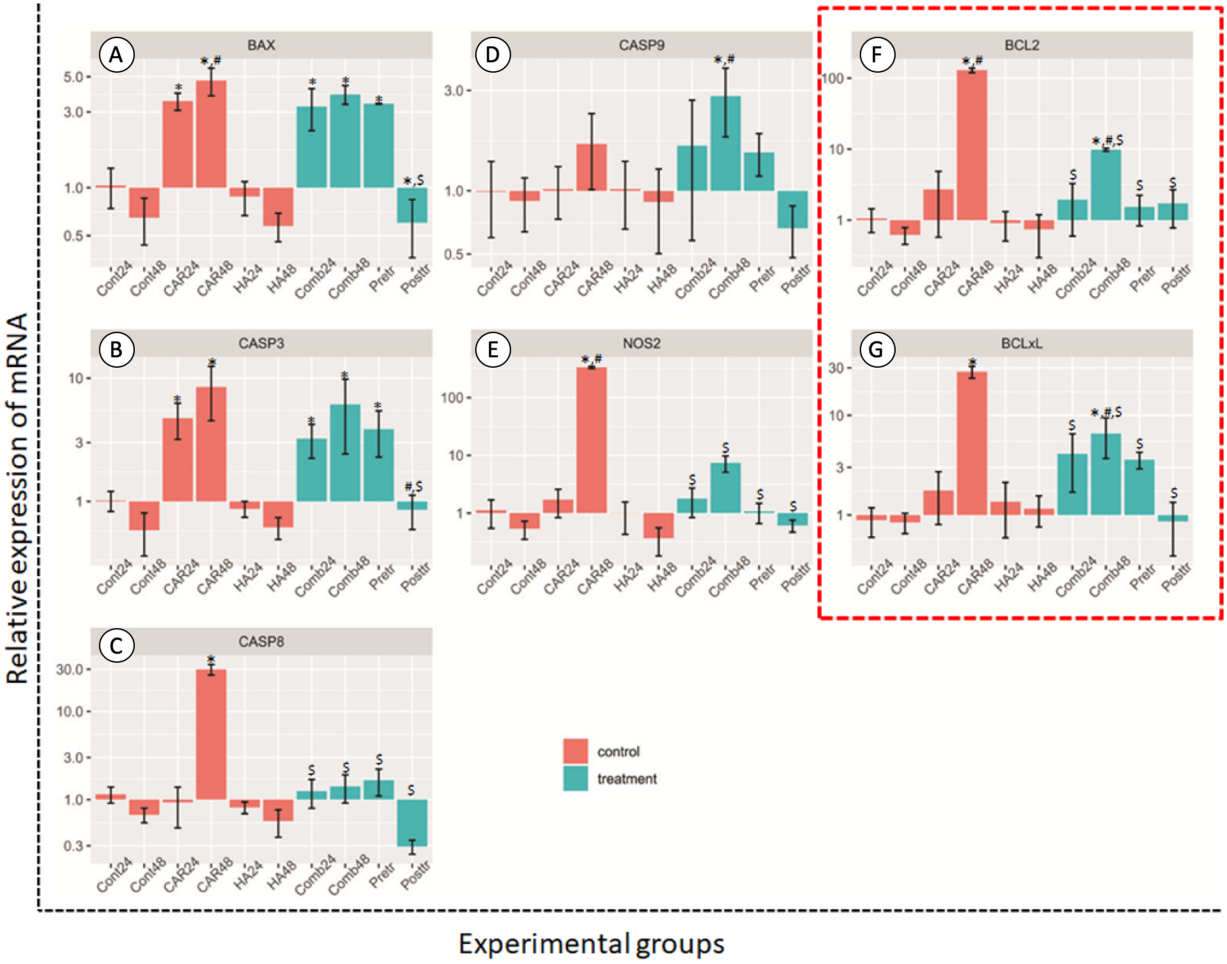
Relative expression (mean ± SD) of five apoptotic genes ((A–E) BCL2 Associated X, Apoptosis Regulator; *BAX*, Caspase 3; *CASP3*, Caspase 8; *CASP8*, Caspase 9; *CASP9* and Nitric oxide synthase 2; *NOS2*) and two anti-apoptotic genes. Insix control (orange bars) and four treatment groups (turquoise bars; F and G). This experiment was performed in six replications and significant differences were observed when *P* < 0.05): “*” represents significant differences when compared with the Cont24 and Cont48 groups, “#” represents significant differences when compared with the CAR24 group and “$” represents significant differences when compared with the CAR48 group.

After treatment, the relative expression levels of the genes as mediators of apoptosis are shown in Fig. 4. The treatment of HA alone at both 24 and 48 h did not induce the up-regulation of chondrocytes in these groups of genes when compared with the control group.

Apoptosis genes; *BAX, CASP3, CASP8* and *NOS2*, and anti-apoptosis genes; *BCL2* and *BCL2-xL* revealed the significantly highest levels of expression in the CAR48 group when compared with the other groups. The expression levels of *BAX* genes in CAR24, CAR48, Comb24, Comb48 and Pre-tr groups were significantly up-regulated when compared with the Cont24 and Cont48 groups. CASP3 was significantly up-regulated in CAR24, CAR48, Comb48 and Pre-tr groups when compared to the Cont24 and Cont48 groups. The expression patterns of *CASP8* and *NOS2* were similar, while the CAR48 group was found to display the significantly highest expression levels when compared with the other nine groups. Additionally, CASP9 expression in Comb48 was significantly higher than the Cont24, Cont48 and CAR24 groups. The expression levels of *BCL2* and *BCL2-xL* were also similar and they were significantly up-regulated in the CAR48 and Comb48 groups when compared with the Cont24, Cont48 and CAR24 groups.

#### Genes related to anabolic and catabolic pathway

**Figure 5:**
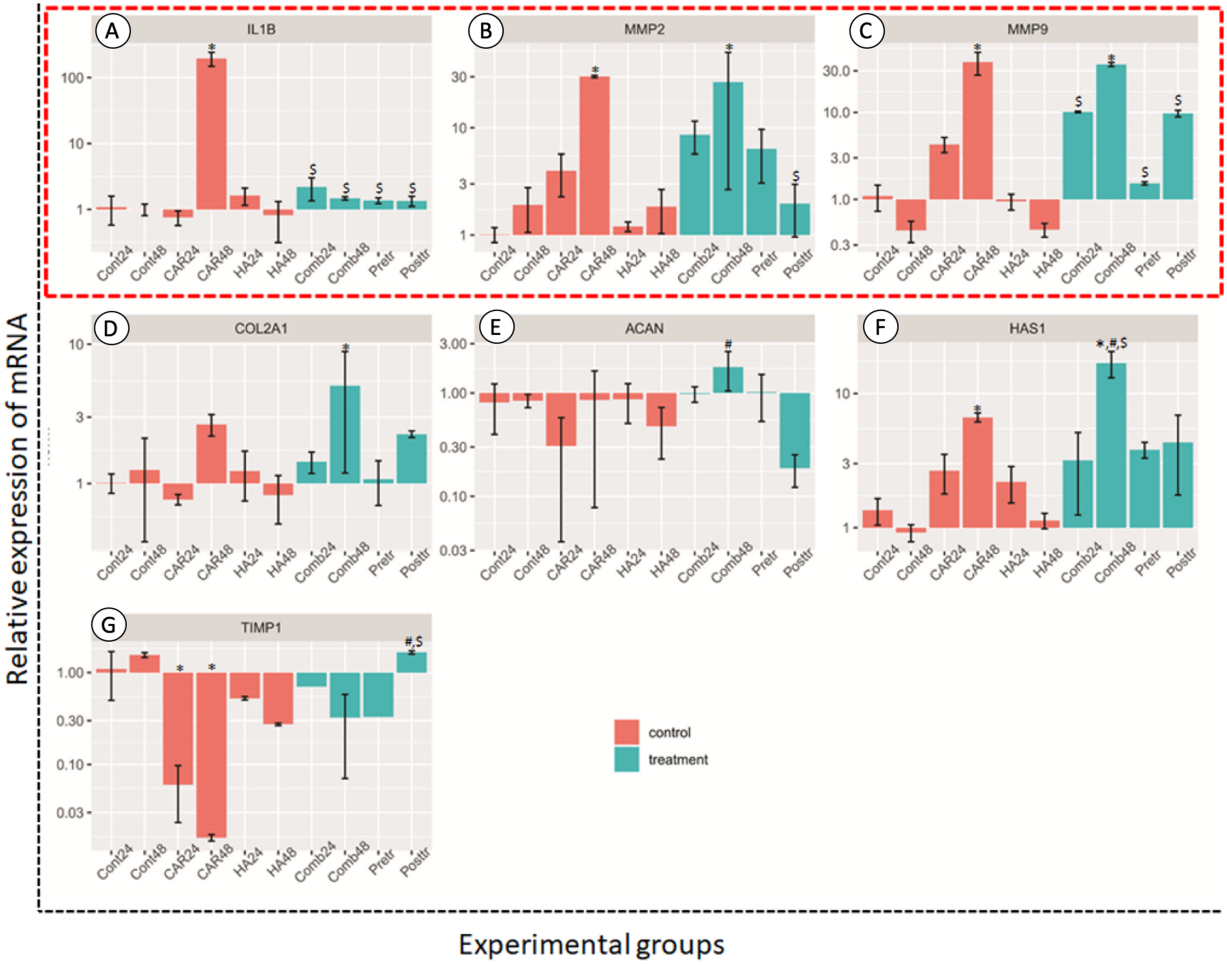
Relative expression (mean ± SD) of four anabolic genes (in the red frame; A–C) and four catabolic genes (D–G) in six control (orange bars) and four treatment groups (turquoise bars). Aggrecan (*ACAN)*, collagen type II alpha 1 Chain (*COL2A1*), hyaluronan synthase 1 *(HAS1)* and TIMP metallopeptidase inhibitor 1 (*TIMP-1*) were used to estimate the effects of the drugs on chondrocyte anabolism. Genes involved with the catabolism of chondrocyte; interleukin-1 Beta (*IL-1*β), matrix metalloproteinase-2 (*MMP2*) and matrix metalloproteinase-9 (*MMP9*) were used to assess the level of expression. This experiment was performed in four replications and significant differences were observed when *P* < 0.05): “*” represents significant differences when compared with the Cont24 and Cont48 groups, “#” represents significant differences when compared with the CAR24 group and “$” represents significant differences when compared with the CAR48 group.

The relative expressions of catabolic genes including *MMP2*, *MMP9* and *IL-1*β, and anabolic genes *ACAN*, *COL2A1*, *HAS1* and *TIMP1*, are shown in Fig. 5. The expression levels of *IL-1*β genes in the CAR48 group were significantly up-regulated when compared with the other nine groups. The expression levels of *MMP2* and *MMP9* in the CAR48 and Comb48 group were significantly up-regulated when compared with the Cont24, Cont48, CAR24, HA24 and HA48 groups. Moreover, *IL-1*β, *MMP2* and *MMP9* expression levels in the Comb24, Pre-tr and Post-tr groups were not significantly different when compared with those of the Cont24, Cont48, HA24 and HA48 groups. No differences of the *ACAN* genes were found in the four treatment groups and the Cont24 and Cont48 groups, but levels were significantly up-regulated in the Comb24 group when compared with the CAR24 group. The expression levels of *COL2A1* genes were found to have been significantly up-regulated in Comb48 when compared with the Cont24, Cont48, CAR24, HA24 and HA48 groups, while the significantly highest expression levels of *HAS1* were found in the Comb48 group when compared with the Control at both 24 and 48 h. Moreover, the expression levels of the genes in the CAR48 group were significantly higher than those of the Cont24 and Cont48 groups. The expression levels of the *TIMP1* gene were down-regulated in the CAR24, CAR48, HA24, HA48, Comb24, Comb48 and Pre-tr except Post-tr groups when compared with the Contrl groups. In addition, the levels of the Post-tr group were significantly up-regulated when compared with those of the CAR24 and CAR48 groups.

## Discussion

Highlights of this study:

1. Post-treatment with HA in normal chondrocyte cells clearly reduced all negative effects after treatment with CAR and had the potential to reduce the side effects of CAR at the transcriptional level to a greater degree than either the pretreatment or the combined treatment.
2. Normal chondrocytes treated with carprofen can decrease cell viability and may induce cell apoptosis via the up-regulation of some apoptotic genes and by inducing the up-regulation of anabolic genes, especially when chondrocytes were exposed to this drug over longer periods of time.
3. Normal chondrocytes treated with hyaluronan did not induce cells to increase cell viability or decrease cell apoptosis. Moreover, the expression levels of the apoptotic, anabolic and catabolic gene were not changed.

Our results revealed that CAR provoked the expression of cartilage-specific catabolic genes including *IL-1*β, *MMP2* and *MMP9*. Additionally, *IL-1*β, the proinflammatory cytokine, plays a key role in the pathogenesis of degenerative joint disease, particularly osteoarthritis (Jenei-Lanzl, Meurer & Zaucke, 2019; Xia et al., 2014). High expressions of this cytokine consequently amplify other processes of arthritis including inflammation, cartilage degradation and the death of chondrocytes (Jenei-Lanzl, Meurer & Zaucke, 2019; Wang & He, 2018). IL-1β induced IL-6 promoted the production of NO via the STAT3 pathway, leading to the induction of the apoptotic process (Wang & He, 2018). Our results confirmed that the side effects of CAR enabled chondrocytes to be attenuated by treatments with hyaluronan, especially in the post treatment. This was shown by the strong suppression of IL-1β gene expression, NO production and the apoptotic related factors. Additionally, the expression of the cartilage-degrading enzymes *MMP2* and *MMP9*, which are generally upregulated by proinflammatory cytokines, seemed to be down-regulated by the post treatment with hyaluronan. This outcome was consistent with the expression levels of *TIMP1*, which could be brought back to the basal level after facing the dramatic suppression by CAR. In addition, the anabolic genes, especially the *HAS1* expression levels, were down-regulated by CAR. This seemed to be a positive response to the treatments with hyaluronan. However, experiments involving animal models are needed for further confirmation.

In accordance with the findings of the previous study, pretreatment with HA before inducing chondrocytes that were exposed to inflammatory cytokines could reduce cell apoptosis in vitro (Grishko et al., 2009; Zhou, Liu & Peng, 2008). Among three different combinations, the post-treatment with HA showed the highest potential for the down-regulation of *BAX, CASP3, CASP8, CASP9* and *NOS2*, along with the anti-apoptosis genes *BCL2* and *BCL2-xL* that were induced by CAR. All of these groups of genes are associated with key apoptotic factors. *CASP3* is a gene that is encoded with the protein caspase-3, the most important of the executioner caspases and one that is activated by any of the initiator caspases (caspase-8, caspase-9, or caspase-10). CASP8 is a caspase protein that is encoded with the *Casp8* gene and is involved in the extrinsic pathway of apoptosis. BAX, a pro-apoptotic member of the Bcl-2 family, is a cell-death effector that directly or indirectly generates the cell death signal, while Bcl-2 is the dominant inhibitor of Bax (Goggs et al., 2003). BCL2 and BCL-xL, as anti-apoptotic members, bind to the multi-domain pro-apoptotic counterparts of Bax to prevent the formation of lethal pores in the outer membrane of the mitochondria, which interrupts the pro-apoptotic signals (Michels et al., 2013). Caspase-9 protein is involved in the intrinsic pathway of apoptosis and then breaks up the cells into apoptotic bodies (Bratton & Salvesen, 2010). These findings confirm that the post-treatment with HA after CAR exposure could decrease cell apoptosis in the normal canine chondrocyte model via both the intrinsic and extrinsic apoptosis pathways.

However, in this study, it was found that the combination treatment with CAR with HA (Comb24 and Comb48) did not decrease chondrotoxicity (and did not increase chondrocyte viability). Similar to our previous study involving OA chondrocyte cultures, co-treatment with HA did not decrease chondrotoxicity caused by CAR (Euppayo et al., 2015), triamcinolone acetonide (Euppayo et al., 2016), dexamethasone (Siengdee et al., 2015) and prednisolone (Siengdee et al., 2015). However, some studies have shown that HA could reduce cytotoxicity on chondrocyte viability after cells were exposed to bupivacaine (Onur, Sitron & Dang, 2013), indomethacin (Hashizume & Mihara, 2009), dexamethasone (Hashizume & Mihara, 2009) and celecoxib (Hashizume & Mihara, 2009). These results might have been caused by the specific properties associated with the molecular weight of HA. Notably, these studies had used a high molecular weight of HA, while our study used a low molecular weight HA. As has been well established, the cartilage matrix consists of three major biomolecules including type II collagen, aggrecan and hyaluronan (Carballo et al., 2017; Xia et al., 2014). *COL2A1* is responsible for the expression of type II collagen and *ACAN* encodes the core protein of aggrecan, the most important proteoglycan of the cartilage matrix (Xia et al., 2014). Hyaluronan, a large glycosaminoglycan found in the network of the cartilage matrix, is produced by hyaluronan synthases, *HAS1, HAS2 and HAS3*. *HAS1* and *HAS2* are responsible for producing high molecular weight hyaluronan, while HAS3 is involved in the production of smaller sizes (Archer & Francis-West, 2003; Bastow et al., 2008). These cartilage-specific anabolic genes, *COL2A1, ACAN* and *HAS1*, were up-regulated by certain growth factors reflecting the matrix-remodeling processes (Carballo et al., 2017; Jenei-Lanzl, Meurer & Zaucke, 2019; Xia et al., 2014). Pro-inflammatory cytokines that cause the pathogenesis of degenerative joint diseases, including IL-1β, IL-6 and TNF-α, suppress the expression of these genes (Xia et al., 2014). In the present study, Comb48 seemed to enhance the expression of these anabolic genes, which may suggest the benefit of HA when combined with CAR. Nevertheless, we have suggested that chondrocytes be more deeply investigated for further use in 3D culture models, such as those involving pellet or scaffold cultures (Kongdang et al., 2019).

This study suggests that long-term CAR exposure could induce chondrocyte apoptosis and may affect the apoptosis pathway and anabolic pathway regulation at the transcriptional level. As was reported in the previous study, NSAIDs could induce apoptosis in various cell types via both the COX-2-dependent pathway and the COX-2-independent pathway (Chan, 2002; Dai & Wang, 2006; Jana, 2008). In the COX-2-dependent pathway, treatment with NSAIDs inhibited COX-2 activity and increased the amount of arachidonic acid (Chan et al., 1998). Accretion of arachidonic acid further stimulated the conversion of sphingomyelin to ceramide via sphingomyelinase and lastly induced apoptosis to a potent degree (Hannun, 1996; Jayadev et al., 1997). In the COX-2-independent pathway, several mechanisms that were reported to have been associated with NSAIDs-induced apoptosis were involved with the COX-2-independent pathway, including cell cycle arrest (Bock et al., 2007; Luciani, Campregher & Gasche, 2007; Piazza et al., 1997), alteration in the levels of pro- and anti-apoptotic proteins (Gu et al., 2005; Ho et al., 2003; Zhou et al., 2001), and activation of the extrinsic and intrinsic pathways of apoptosis (Bellosillo et al., 1998; Deng et al., 2009; Redlak, Power & Miller, 2005; Zimmermann et al., 2000). Some reports have shown that NSAIDs displayed an anti-apoptosis mechanism by blocking NO and de-differentiating the rabbit articular chondrocytes by the modulation of ERK, p38 kinase and PKCα and –ζ (Yoon et al., 2003). This study found that the treatment at 0.16 mg/mL CAR either at 24 or 48 h could cause chondrocyte apoptosis to be observed via chromatin condensation, DNA fragmentation, and the apoptotic bodies (karyorhexis) that were presumably involved in the COX-2-independent pathway at a molecular level by activating the up-regulation of some apoptotic genes (*BAX*, *CASP3*, *CASP9*) and by inducing NO production. However, we did not observe chondrocyte apoptosis to be involved with the COX-2-dependent pathway. Further studies should help to confirm this point and provide important information on the involvement of the effects of NSAIDs on the chondrocyte apoptosis pathways. Apart from the apoptosis pathway, the present study indicated that a prolonged exposure of chondrocytes with CAR may lead to cartilage degradation, as can be observed in the induction of catabolic genes; *IL-1*β, *MMP2* and *MMP9* and the suppression of anabolic genes; *ACAN* and *TIMP1*. In particular, the highest expression of *IL-1*β, which is well-established as the key cytokine for the cartilage degradation pathway (Jenei-Lanzl, Meurer & Zaucke, 2019). Our results also suggest that treatments by the combination of CAR and hyaluronan following the administration with hyaluronan alone for some period of time may relieve the chondrotoxicity of CAR and allow for the process of cartilage remodeling.

## Conclusion

Carprofen treatment could decrease cell viability and may induce cell apoptosis and the up-regulation of some apoptotic genes, as well as to induce the up-regulation of anabolic genes. This is especially true during treatments over long periods of time. A combined treatment of HA and CAR for 48 h was effective in inducing chondrocytes in the down-regulation of *NOS2, CASP8, BCL2* and *BCL2-xL*, as well as the up-regulation of catabolic genes. Post-treatments with HA (incubated CAR 24 h and then replaced by HA 24 h) in normal chondrocytes had the highest potential to reduce all of the negative effects after the treatment of CAR, including decreased apoptotic levels, decreased NO production, the down-regulation of the apoptosis genes, and some anabolic genes in the chondrocytes. This result has revealed the potential benefits of the anti-apoptosis effects of HA post-treatment after being induced with CAR in normal canine chondrocytes in vitro.

## Supplemental Information

10.7717/peerj.8355/supp-1 Supplemental Information 1 Raw data.

## Additional Information and Declarations

### Data Availability

The following information was supplied regarding data availability: The raw data are available as a Supplemental Files.
